# FGF23^C-tail^ improves diabetic nephropathy by attenuating renal fibrosis and inflammation

**DOI:** 10.1186/s12896-018-0449-7

**Published:** 2018-05-30

**Authors:** Xiaomin Zhang, Kaiwen Guo, Feng Xia, Xinyu Zhao, Zhifeng Huang, Jianlou Niu

**Affiliations:** Department of Biopharmacy, School of Pharmacy, Wenzhou Medical University, Chashan Town, Wenzhou, 325035 China

**Keywords:** FGF23, Diabetic nephropathy, Inflammation, Fibrosis

## Abstract

**Background:**

High level of serum fibroblast growth factor 23 (FGF23) is implicated in the development and progression of diabetic nephropathy (DN), making it a crucial factor in the pathogenesis of DN. FGF23 is also tightly correlated with inflammation in the progression of DN. The aim of this study was to explore whether the C-terminal of FGF23 (FGF23^C-tail^), an antagonist that can block the FGF23 signaling pathway by competing with intact FGF23, could exhibit a therapeutic effect on DN.

**Results:**

Biochemical data and histological examination showed that FGF23 ^C-tail^ administration ameliorated the functional and morphological abnormalities of db/db mice with DN without changing the levels of circulating FGF23 and phosphate. Evaluation of morphology and fibrosis by Masson’s trichrome staining and IHC staining of fibronectin, PCR, and western blot analysis showed that FGF23^C-tail^ prevents diabetes-induced fibrosis in db/db mice. Importantly, FGF23^C-tail^ decreased the levels of inflammatory cytokines in serum and renal tissues.

**Conclusion:**

FGF23^C-tail^ may improve diabetic nephropathy by decreasing inflammation and fibrosis in db/db mice, suggesting that blocking of FGF23 action remains an important therapeutic target for the prevention or attenuation of the progression of DN.

**Electronic supplementary material:**

The online version of this article (10.1186/s12896-018-0449-7) contains supplementary material, which is available to authorized users.

## Background

Diabetic nephropathy (DN) is one of the major causes of end-stage renal disease in diabetic patients in developed countries, mainly due to the increasing prevalence of type 2 diabetes [1, 2]. DN is characterized by proteinuria, glomerular hypertrophy, thickened basement membrane, podocytopenia, increased extracellular matrix protein deposition, and fibrosis [3]. The mechanism involved in diabetes-induced renal disease is complex. Emerging evidence suggests that inflammatory mechanisms play an important role in the etiology of DN [4]. Notably, with relevance to our research, studies of the pathophysiology of DN found that the level of fibroblast growth factor 23 (FGF23) in plasma was significantly upregulated in DN, and the circulating level of FGF23 continued to increase with the further progression of DN [5–7]. Abundant clinical and experimental evidence indicate a positive association of circulating FGF23’s levels and the stage of DN [8–10]. Consistent with these findings, recent research reported that the plasma FGF23 concentration is a risk factor of diabetic nephropathy (DN) triggering a series of pathological changes in kidney, and a valuable and sensitive biomarker in several acute and chronic disorders [11–14]. In the past decades, a significant body of evidence has demonstrated that gradual elevation of FGF23 over time can result in inflammation in patients with diabetes-induced nephropathy [15, 16]. Therefore, to explore strategies that block FGF23 action is an important therapeutic target in preventing or attenuating the progression of DN and the occurrence of relative complications.

Fibroblast growth factors (FGFs) are a family of polypeptides with diverse biological functions related to cellular development, differentiation, migration, and repair [17]. FGF23 belongs to the endocrine subfamily of FGFs that also includes FGF19 and FGF21. A multitude of studies have demonstrated that FGF23 is a secretory molecule that is mainly produced by osteoblastic cells and was originally shown to function as a central regulator of phosphate (Pi) and vitamin D metabolism [18]. FGF23 activity is regulated by proteolytic cleavage at the ^176^RXXR^179^ motif, located at the boundary between the FGF core homology domain and the 72-residue-long C-terminal tail of FGF23 (FGF23^C-tail^) [19]. This unique C-terminal sequence mediates high affinity binding to the FGFR/Klotho receptor complex [20, 21]. Previous study demonstrated that the isolated FGF23^C-tail^ could interfere with FGF23 signaling by competing with the full-length ligand for binding to the binary FGFR-Klotho complex, making FGF23^C-tail^ a promising therapeutic agent for the treatment of diseases in which the overexpression and accumulation of FGF23 induced the further progression of disease [20].

In the present study, we investigated the hypothesis that the FGF23^C-tail^ could ameliorate the development of DN as a competing antagonist of intact FGF23 in mouse models of T2D (db/db). Our findings indicated that the use of the FGF23^C-tail^ significantly improved renal dysfunction and morphologic damage by reducing fibrosis and renal inflammation, although no significant decrease was observed in the plasma level of FGF23 and hyperglycemia was not corrected. To our knowledge, this is the first demonstration of the protective effect of FGF23^C-tail^ on diabetic nephropathy.

## Methods

### Material

FGF23^C-tail^ was produced by the Key Laboratory of Biotechnology and Pharmaceutical Engineering of Zhejiang Province, Wenzhou Medical University.

Antibodies against CD68, IL-6, fibronectin, and collagen IV were produced by Abcam (Cambridge, UK), and antibodies against MCP-1 and GAPDH were purchased from Cell Signaling Technology (CST, USA). QuantiQuik™ Urea (BUN) Quick Test strips for blood urea nitrogen (BUN) in urine and kits for serum creatinine were purchased from Bioassay Systems (CA, USA). The ELISA kit for microalbumin in urine was from Abcam (Cambridge, UK). ELISA kits for pro-inflammatory cytokines (IL-6, TNFα, MCP-1, and IP-10) were obtained by Thermo Fisher Scientific (NY, USA). The kit for reverse transcription and the SYBR mix was purchased from Invitrogen (CA, USA). All other chemicals and reagents used in these experiments were of analytical grade.

### Animal experiments

**Fig. 1:**
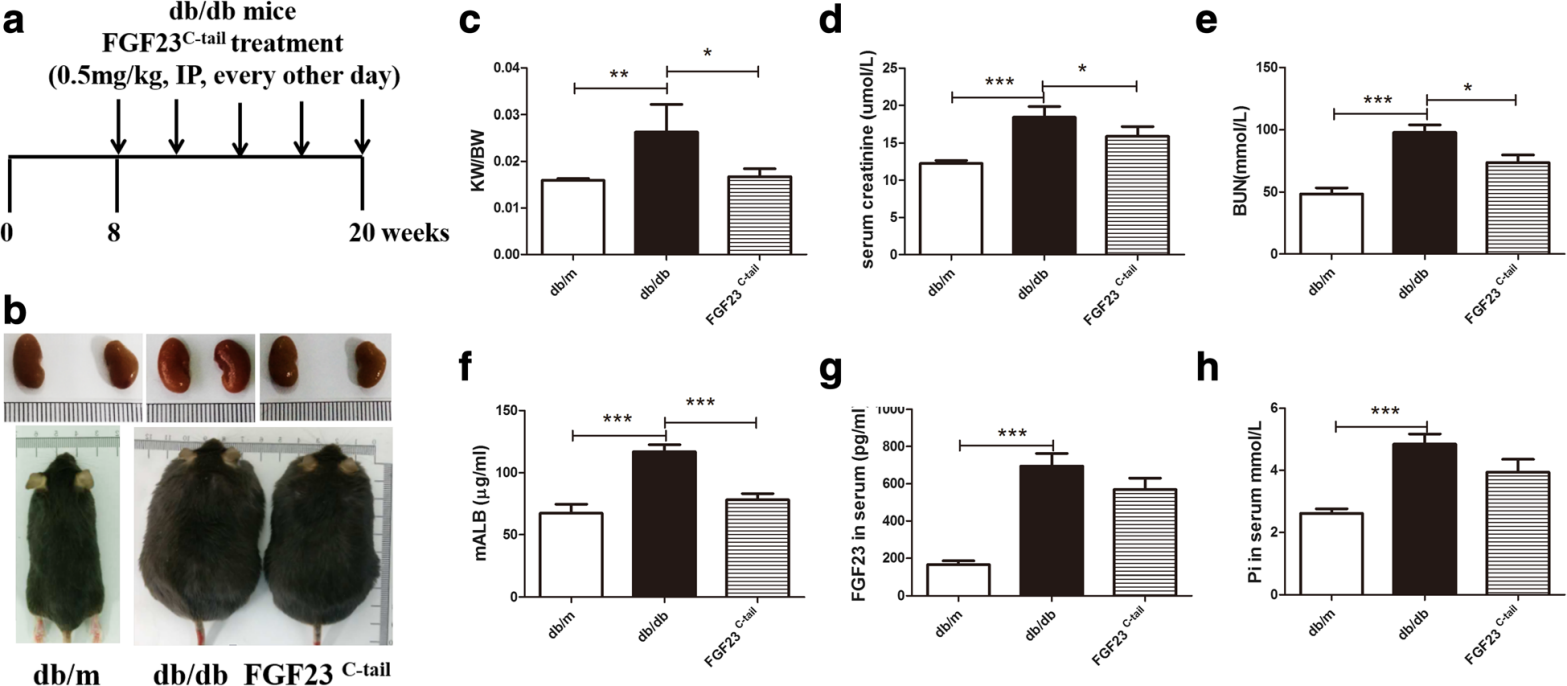
FGF23^C-tail^ treatment improved renal hypertrophy and dysfunction in db/db mice. **a** Treatment protocol for db/db mice wherein db/db mice received vehicle or FGF23^C-tail^ (0.5 mg/kg body weight (BW), every other day) for 12 weeks; **b** Photograph of representative mice and kidneys of db/m, db/db, and FGF23^C-tail^ group. **c** Kidney weight-to-body weight (KW/BW) ratio in db/m, db/db, and FGF23^C-tail^ group; **d** Renal function was determined by measuring excretion of creatinine and **e** the level of BUN and **f** micro-albumin in urine; **g** The levels of FGF23 in serum; **h** the levels of phosphorous (Pi) in serum

C57BLKS/J-Leprdb/Leprdb (db/db) and C57BLKS/J-Leprdb/m (db/m) male mice were obtained from the Model Animal Research Center of Nanjing University (Nanjing, China) for this study. The db/db mice were randomly and equally divided into two groups: vehicle group (*n* = 10) and FGF23^C-tail^ group (*n* = 10). The vehicle group and FGF23^C-tail^ group (*n* = 10) were treated with PBS or FGF23^C-tail^ (0.5 mg/kg), respectively, by intraperitoneal injection every other day for 12 weeks, as shown in Fig. 1a. The db/m littermates (*n* = 10) were treated with PBS, and served as the sham group. All mice were housed under controlled conditions with free access to food and water. Blood glucose levels were monitored by using the Precision G Blood Glucose Testing System (Abbott Laboratories, Abbott Park, IL) and the body weight was recorded. After 12 weeks of treatment, all mice were kept in individual metabolic cages to allow 24 h of urine collection and then the blood samples were collected. After that the animals were sacrificed, kidney tissues were harvested for subsequent studies. BUN and microalbumin in urine, serum creatinine and proflammatory cytokines (IL-6, TNF-α, MCP-1 and IP-10) were measured by ELISA Kit according to the manufacture’s instructions. All experiments were conducted in accordance with the National Institutes of Health guidelines and with approval of the Wenzhou Medical University Institutional Animal Care and Use Committee.

### Histopathological examination

The harvested kidney tissue samples were fixed overnight in 4% paraformaldehyde. Then, the tissues were embedded in paraffin, and then the paraffin blocks were cut into 5-μm pieces and transferred to slides. After deparaffinization and rehydration, the 5 μm-thick serial sections were stained with hematoxylin and eosin (HE) for routine histopathological observations. Periodic acid-Schiff (PAS) and Masson’s trichrome staining was performed to determine collagen deposition and fibrosis. All of these sections were analyzed to evaluate tubular and glomerular injury.

### Immunofluorescence staining and immunohistochemistry

Immunofluorescence (IF) Staining and Immunohistochemistry (IHC) were performed on 5-μm paraffin-embedded kidney sections. After deparaffinization and rehydration, the slides were placed in citric acid buffer and microwaved for 3 min at 900 W, 150 W for 8 min, followed by cooling for 30–60 min at room temperature. Next, all kidney slides were treated with 3% H_2_O_2_ for 30 min and then blocked with 5% BSA for 60 min at 37 °C. The slides were then incubated overnight with primary antibodies at 4 °C in a wet box, using an HRP-conjugated goat anti-rabbit or mouse secondary antibody. For Immunofluorescence Staining, we used a secondary antibody conjugated to FITC and the nuclei were stained using 40,6-diamidino-2-phenylindole (DAPI). Sections in the slides were examined using an inverted Leica DM inverted microscope and SPOT camera system.

### Transmission electron microscopy

The morphological changes of renal tissue were observed by transmission electron microscopy (TEM) as done previously [22]. Briefly, the kidney tissues were fixed, dehydrated, and sealed with an acetone/resin mixture in an oven at 60 °C for 24 h. Then, the kidney tissues were cut into semi-thin sections (1 μm) and stained with toluidine blue. Later, the samples were cut into ultra-thin sections, stained with lead nitrate and uranyl acetate, and then examined with a transmission electron microscope H7500 (Hitachi, Tokyo, Japan).

### Real-time PCR

Total RNA samples were isolated from renal tissue using an RNA extraction kit (Beijing Tiangen Biotech, China). Real-time RT-PCR was performed using a 2-step M-MLV Platinum SYBR Green qPCR SuperMix-UDG kit (Invitrogen). All kits were used strictly according to the manufacturer’s instructions. Amplification of glyceraldehyde 3-phosphate dehydrogenase (GAPDH) was performed as an endogenous control to normalize the amount of total RNA in each reaction and the relative expression of target mRNA was calculated according to the − 2∆∆Ct method.

### Western blot analysis

Proteins were extracted from mice renal tissue using RIPA buffer supplemented with protease and phosphatase inhibitors. The amount of total protein was detected using a BCA protein assay kit, and homogenates with equal amount of proteins were subjected to SDS-polyacrylamide gel electrophoresis and then transferred to polyvinylidene difluoride (PVDF) membranes. The blots were blocked with 10% milk in PBS for 1 h at room temperature, and then incubated with the primary antibodies for 12 h at 4 °C, washed and treated with PBST and HRP-conjugated secondary antibodies. Finally, chemiluminescence (ECL) was used to detect the bands when exposed to X-ray film.

## Results and Discussion

### FGF23 ^C-tail^ administration improved renal functions in db/db mice

The db/db mouse, a genetic model of type 2 diabetes, has been characterized as a model for obesity, sustained hyperglycemia, and hyperinsulinemia [23–25]. Recently, this animal model has been reported to present with severe diabetes-induced renal changes that are characterized by increased renal/glomerular volume, renal hypotrophy, a high level of creatinine in plasma, and the abnormal excretion of microalbumin and BUN in urine [26–28]. Therefore, db/db mice were ideal to evaluate the therapeutic effect of FGF23^C-tail^ on diabetic nephropathy. We tried 0.1 and 0.5 and 2.5 mg/kg FGF23^C-tail^ in the db/db mice and investigated the effects of different doses of FGF23^C-tail^ on the renal-function using three biomarkers (serum creatinine, blood urea nitrogen (BUN), and albumin). The results showed that 0.5 mg/kg FGF23^C-tail^ showed the best therapeutic effects on renal-function in DN mice (Additional file 1 Figure S1).

After 12 weeks of treatment with 0.5 mg/kg FGF23^C-tail^, plasma glucose levels, body weight, renal morphology, and the kidney weight/body weight (KW/BW) ratio were analyzed. The results showed that FGF23^C-tail^ treatment did not change body weight or glucose levels (data not shown), however, morphological changes of renal hypertrophy in db/db mice were significantly improved in the FGF23^C-tail^ group (Fig. 1b, c). The renal functions were evaluated by determining the levels of three biomarkers of renal injuries (serum creatinine, blood urea nitrogen (BUN), and albumin). The levels of serum creatinine, blood urea nitrogen (BUN), and albumin in db/db mice were much higher than the levels in db/m mice, which indicated a severe decline of renal functions. However, the increases in these biomarkers were significantly attenuated by FGF23^C-tail^ administration, suggesting that the treatment with FGF23^C-tail^ could improve the renal functions of DN (Fig. 1d, f).

Clinical studies observed markedly elevated levels of intact FGF23 and phosphorous (Pi) in serum in diabetic patients with nephropathy [29, 30], suggesting these two factors can serve as biomarkers to predict the progression of diabetic nephropathy [30, 31]. In this study, we also examined the levels of intact FGF23 and phosphate in serum, and found that compared with db/db mice, the plasma levels of FGF23 and phosphate in the FGF23^C-tail^ group were slightly decreased, but not significantly different, suggesting the observed therapeutic effect was independent of the levels of circulating FGF23 and phosphorous (Pi) (Fig. 1g, h).

### FGF23 ^C-tail^ improved renal glomerular lesions in db/db mice

**Fig. 2:**
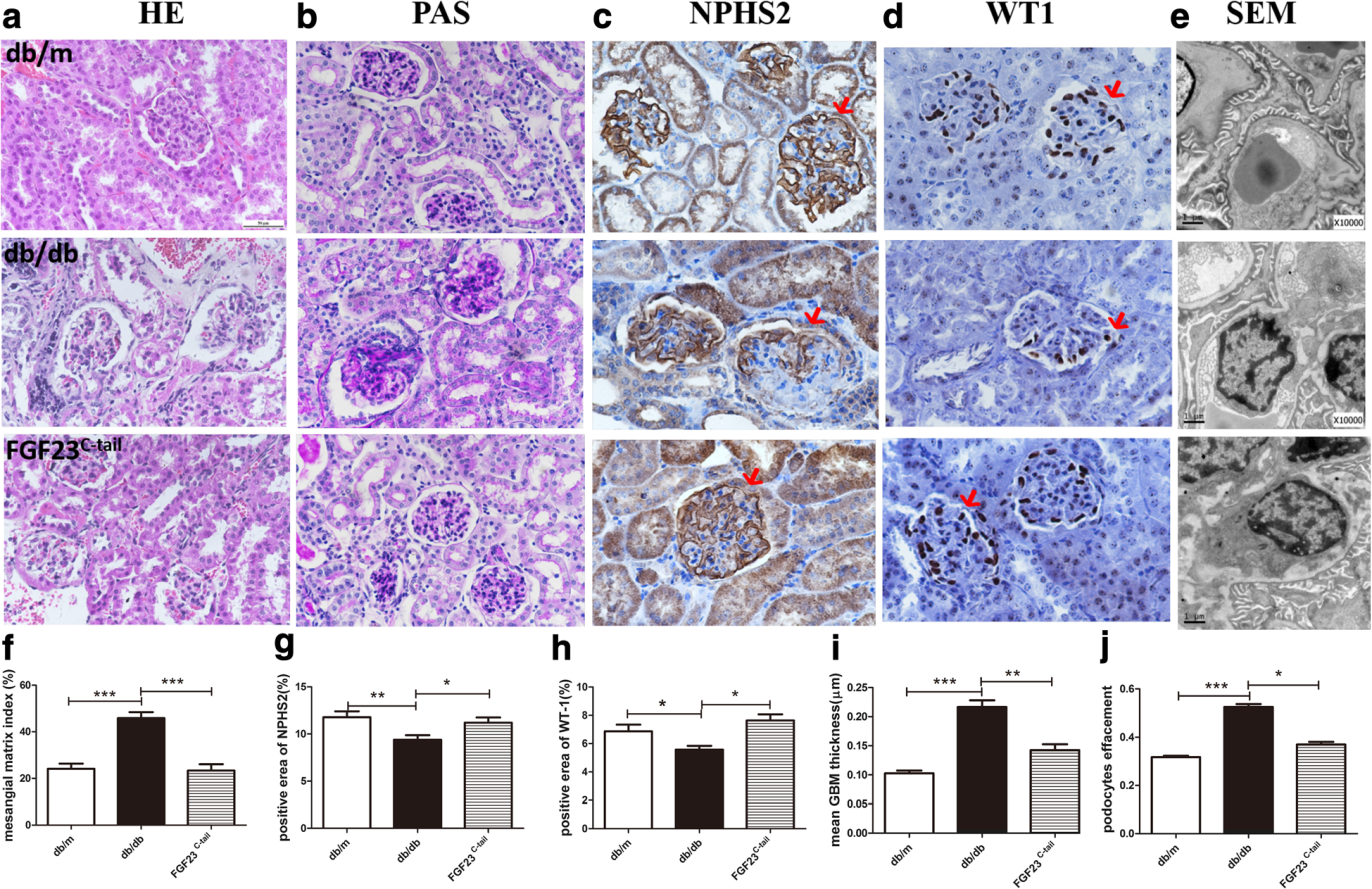
FGF23^C-tail^ treatment strikingly improved renal glomerular lesions in db/db Mice. **a** H & E staining of renal tissues; **b** PAS staining (indicating tubular injury and mesangial expansion; **c-d** Immunochemistry for NPHS2 and WT1, two biomarkers of podocytes; **e** Electron microscope images to observe the morphology of podocytes. Original magnification ×10000; **f** Mesangial matrix index statistically obtained from Panel B; **g-h** Semi-quantitative analysis for the NPHS2 and WT1-positive area; **i-j** Mean GBM thickness and podocyte effacement were semi-quantitatively analyzed based on Panel **e**

Previous reports showed that the deposition of collagen in glomerulars has a central role in renal injury and may contribute to the development of diabetic kidney disease. Thus, hematoxylin and eosin (HE) and periodic acid–Schiff staining (PAS) were performed to examine the effects of FGF23^C-tail^ on the histologic changes and collagen deposition in kidneys in db/db mice. Consistent with improved renal function with treatment with FGF23^C-tail^, the data demonstrated that compared with db/db mice, marked glomerular sclerosis, mesangial expansion, and collagen deposition in kidney sections were prevented by FGF23^C-tail^ treatment (Fig. 2a, b and Fig. 2f). Podocytes are associated with increased susceptibility to kidney injury after exposure to sustained hyperglycemia, and the loss or apoptosis of podocytes has been implicated in the occurrence of proteinuria [32–34]. Therefore, we next investigated the protective effect of FGF23^C-tail^ in podocyte damage. We conducted immunochemistry (IHC) of NPHS2 and WT1, bio-markers of podocytes, to measure the loss of podocytes (Fig. 2c, d). We also performed transmission electron microscopy (TEM) to observe the infusion and effacement of podocytes, as well as the thickening of the basement membrane (Fig. 2e). Semi-quantitative analysis of IHC staining and TEM results revealed significant depletion of podocytes and thickening basement membrane in db/db mice. Interestingly, administration of FGF23^C-tail^ improved podocyte damages (Fig. 2g-j). These data suggested that FGF23^C-tail^ provided therapeutic effect in slowing the progression of diabetic nephropathy via the protection of podocytes.

### FGF23 ^C-tail^ prevents diabetes-induced fibrosis in db/db mice

**Fig. 3:**
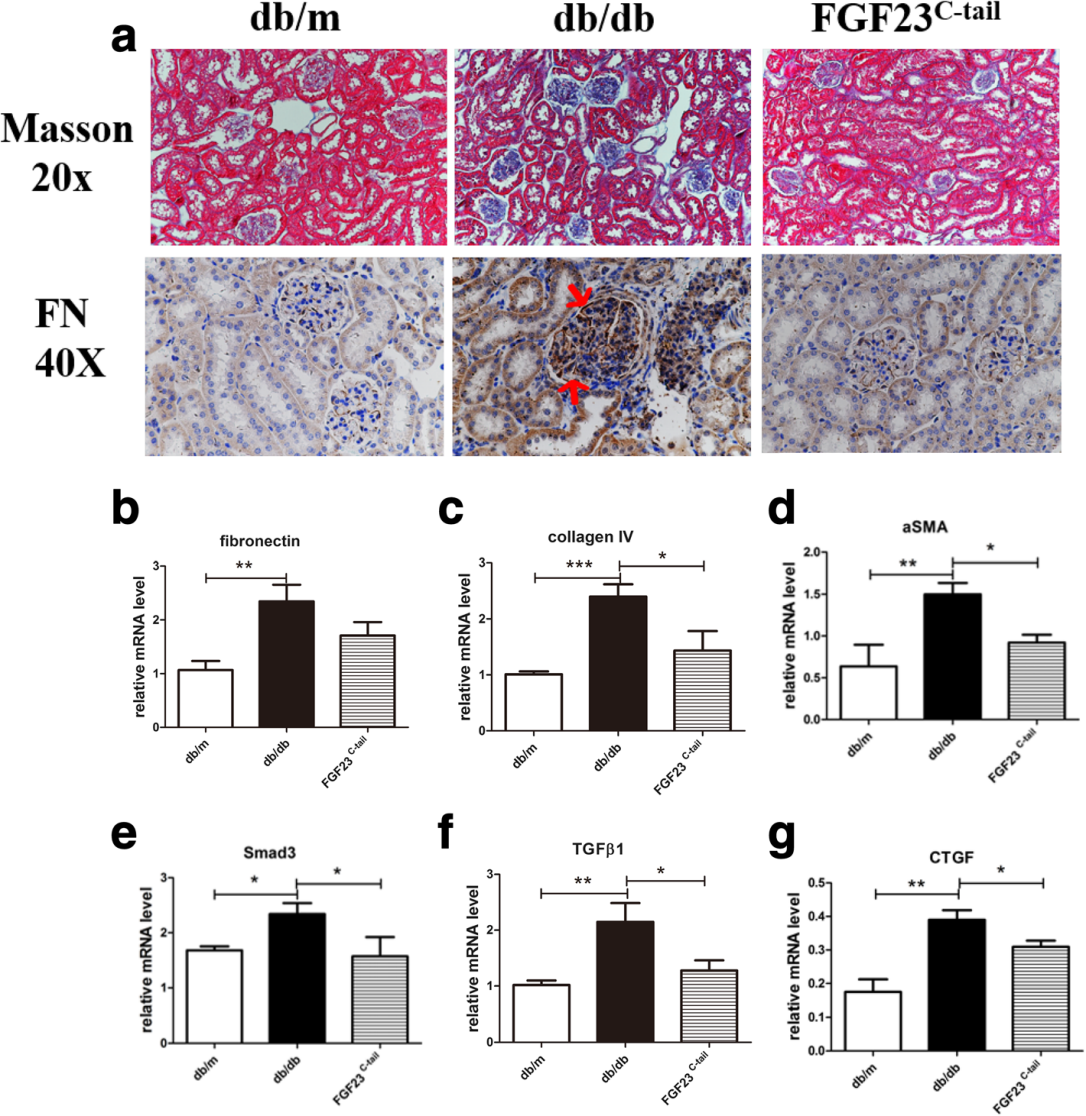
FGF23^C-tail^ administration improved diabetes-induced fibrosis in db/db mice. **a** Masson’s trichrome staining and IHC staining of fibronectin were performed to assess renal fibrosis, arrows indicate the extracellular matrix; **b-g** The relative mRNA levels of pro-fibrotic genes, including fibronectin, collagen IV, αSMA, Smad3, TGF-β1 and CTGF, as analyzed by RT-PCR

DN is characterized by a pronounced collagen deposition and glomerular sclerosis, which facilitated the development of renal fibrotic lesions [35]. Thus, the progression of the fibrosis plays a fundamental role in diabetic nephropathy, and the abrogation or improvement of fibrosis may alleviate DN. In the present study, we investigated the effect of FGF23^C-tail^ administration on fibrosis. Renal glomerular fibrosis was examined by Masson’s trichrome staining and IHC staining of fibronectin was used to evaluate morphology and fibrosis. The results indicated that compared with db/db mice, mice that received FGF23^C-tail^ treatment exhibited improved diabetes-induced fibrosis, suggesting that the effect of FGF23^C-tail^ on DN may be anti-fibrotic (Fig. 3a). To investigate this further, we conducted PCR analysis to measure the expression of genes related to fibrosis. Compared with db/m mice, the levels of profibrotic genes, including fibronectin, collagen IV, α-smooth muscle actin (αSMA), Smad3, transforming growth factor-beta 1 (TGF-β1), and connective tissue growth factor (CTGF) in kidneys were upregulated in db/db mice. However, the up-regulation of these genes were significantly down-regulated by FGF23^C-tail^ administration (Fig. 3b-g).

**Fig. 4:**
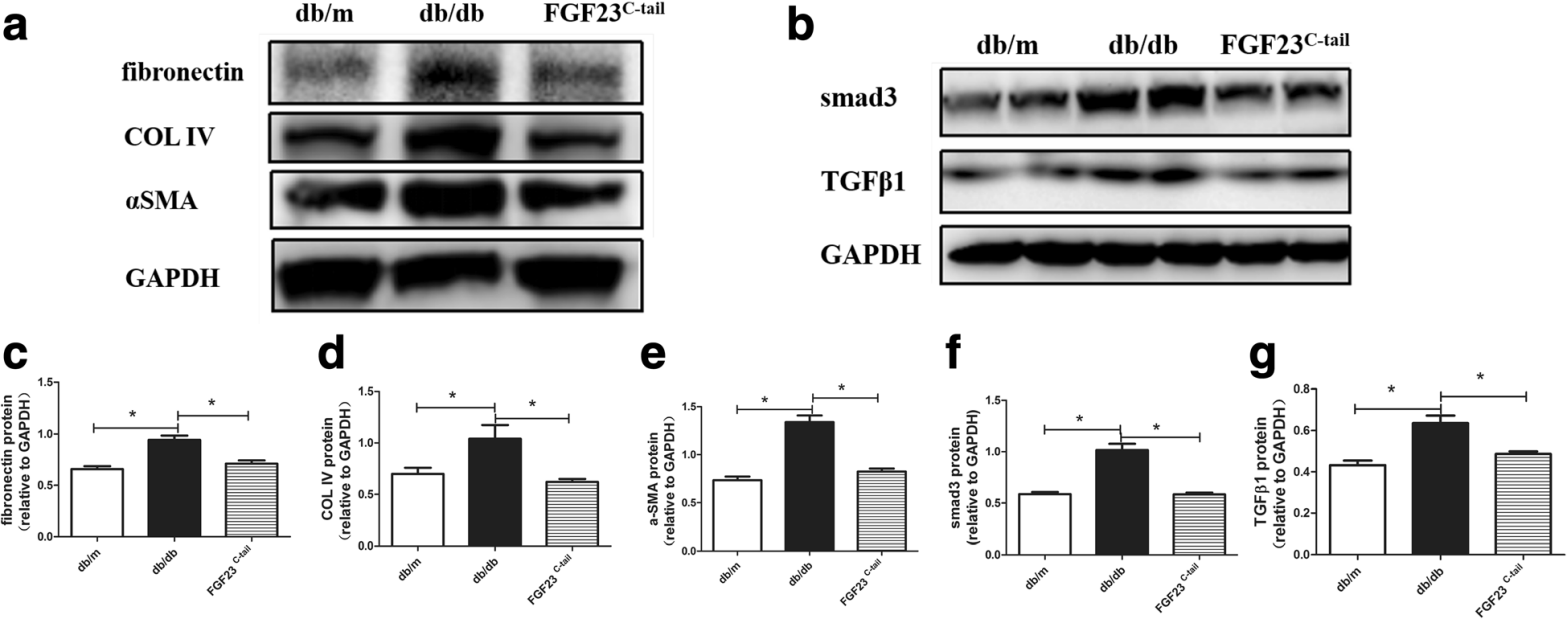
The effects of FGF23^C-tail^ administration on the levels of profibrotic proteins in kidneys. **a-b** Western blot analysis of fibronectin, COL IV, αSMA, Smad3, and TGF-β1; **c-g** Semi-quantitative western blot analysis of the data in panel A & B, GAPDH expression was used for the normalization of protein loading

To further confirm that FGF23^C-tail^ is effective against type 2 diabetes-induced renal fibrosis, western blot analysis was performed to determine the effect of FGF23^C-tail^ administration on the levels of profibrotic proteins in kidneys (Fig. 4a, b). Consistent with the above analysis of profibrotic gene expression, semi-quantitative western blot analysis of fibronectin, COL IV, αSMA, Smad3 and TGF-β1 showed that compared with the db/m mice, the levels of these fibrosis-related proteins were markedly increased in db/db mice, suggesting that db/db mice exhibited more severe collagen deposition and FGF23^C-tail^ could suppress the expression of these diabetes-induced fibrosis-related proteins (Fig. 4c-g).

In summary, the evaluation of fibrosis in renal tissue and the expression of profibrotic genes and proteins confirmed that FGF23^C-tail^ showed renoprotective effect by preventing the progression of fibrosis.

### FGF23^C-tail^ decreased the levels of inflammatory cytokines in serum

**Fig. 5:**
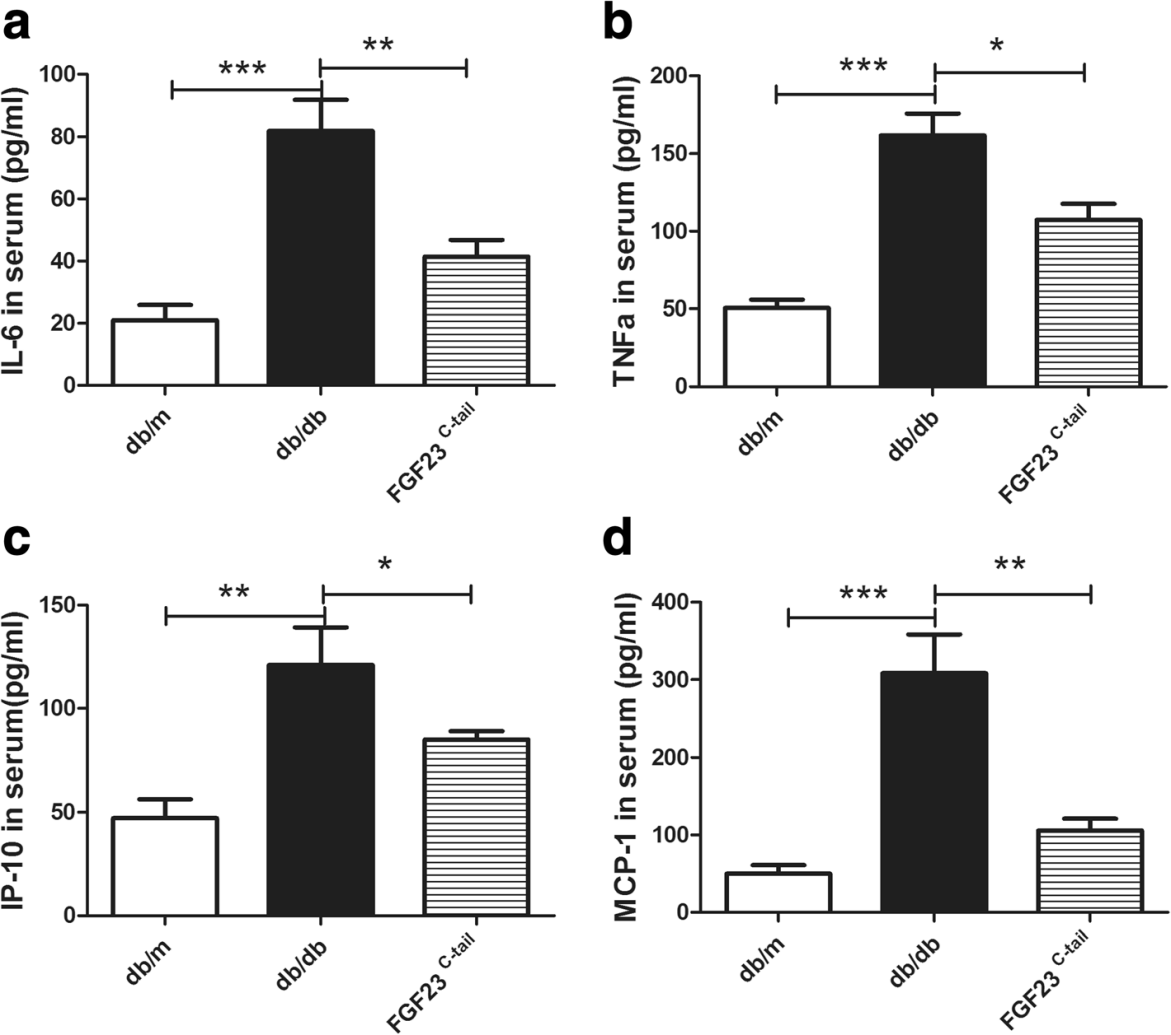
FGF23^C-tail^ decreased the levels of inflammatory cytokines in serum in db/db mice. ELISA analysis of IL-6 **a**, TNFα **b**, IP-10 **c**, MCP-1 **d** protein levels in serum collected from db/m, db/db, and FGF23^C-tail^ group

Inflammation is implicated in the development and progression of diabetic nephropathy, and is believed to be a crucial factor in the pathogenesis of DN and associated with adverse outcomes in many clinical settings [36–38]. The alleviation or inhibition of the occurrence or development of inflammation is a potential target for the treatment of diabetic nephropathy. Thus, we investigated the plasma levels of pro-inflammatory cytokines interleukin-6 (IL-6), tumor necrosis factor α (TNF-α), monocyte chemotactic peptide-1 (MCP-1), and interferon-inducible protein-10 (IP-10), secreted from either inflammatory cells or renal resident cells. The levels of plasma pro-inflammatory cytokines in db/db mice were much higher than those of db/m mice. However, the observed amplification of these pro-inflammatory factors induced by diabetes was remarkably abrogated by the FGF23^C-tail^ treatment, suggesting that FGF23 may protect against diabetic nephropathy by inhibiting inflammation (Fig. 5a-d).

### FGF23^C-tail^ decreased the levels of inflammatory cytokines in renal tissues

**Fig. 6:**
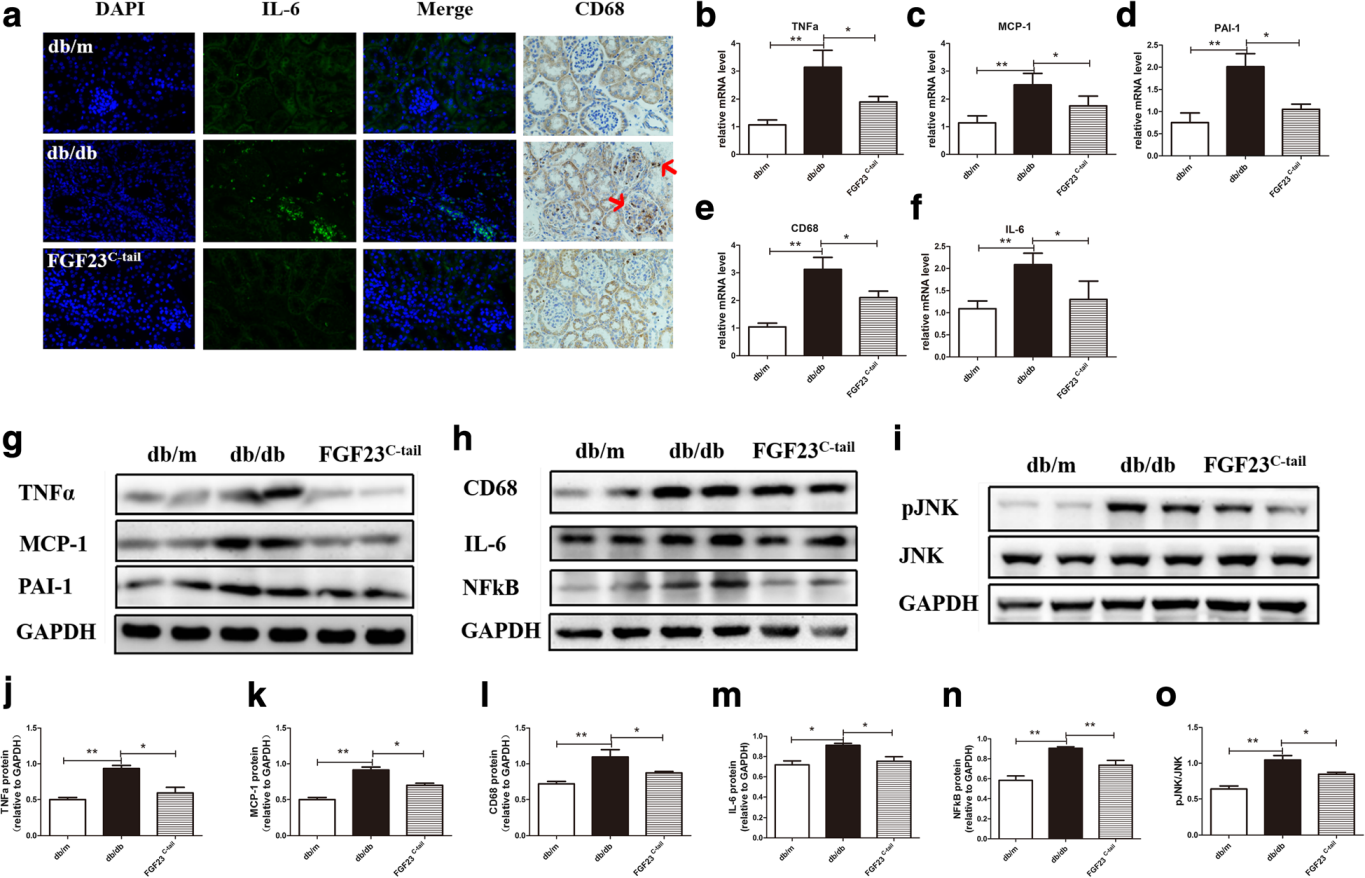
Inflammation in renal tissue induced by diabetes was partly prevented by FGF23^C-tail^ in db/db mice. **a** IF analysis of renal IL-6 expression (original magnification 200×) and IHC analysis of renal CD68 expression (original magnification 400×); **b**-**f** Relative renal levels of TNFα, MCP-1, PAI-1, CD68, and IL-6 mRNAs in db/m, db/db, and FGF23^C-tail^ group. Representative western blots for TNFα, MCP-1, and PAI-1 **g** and CD68, IL-6, and NFkB **h** pJNK/JNK **i**, in lysates of renal tissues from db/m, db/db, and FGF23^C-tail^ group and semi-quantitation for corresponding proteins **j**-**o**

In order to investigate the effect of FGF23^C-tail^ administration on inflammation in the renal tissues of db/db mice, we next determined the levels of two typical inflammatory markers, IL-6 and CD68, in the kidneys. As shown in Fig. 6a, the renal expression levels of IL-6 and CD68 were increased in db/db mice and were greatly suppressed by FGF23^C-tail^ treatment. Consistent with the immuno-staining of kidney sections, the mRNA levels of pro-inflammatory genes, including TNF-a, MCP-1, plasminogen activator inhibitor-1 (PA1–1), CD68 and IL-6, were upregulated in db/db mice. However, the upregulation of these genes was downregulated by FGF23^C-tail^ treatment (Fig. 6b-f). Consistent with the changes of these pro-inflammatory genes, western blot confirmed that the levels of pro-inflammatory proteins induced by diabetes in db/db mice were markedly reduced by FGF23^C-tail^ treatment (Fig. 6g, h). Moreover, we determined that the expression of NF-kB, a key regulator of inflammation that controls the expression of hundreds of pro-inflammatory genes, and the phosphorylation of JNK, another master regulator of inflammation and an important upstream regulator of transcription factors in a variety of cell types [39–41] were increased in db/db mice, and were inhibited by FGF23^C-tail^ treatment (Fig. 6h, i).

The above data were consistent with previous observation of increased expression of cytokines in DN and marked activation of the NFκB pathway in kidneys from human DN and mouse disease models. Overall, FGF23^C-tail^ exerts direct or indirect effects on inflammation to protect kidneys from diabetes-induced nephropathy.

## Conclusion

In conclusion, we found that FGF23^C-tail^, as a competing antagonist of intact FGF23, ameliorates the development of diabetic nephropathy by improving renal dysfunction and morphologic abnormality. This therapeutic effect for the treatment of diabetic nephropathy in db/db is due to the reduction of fibrosis and inflammation resulting from FGF23^C-tail^ treatment. These findings suggest that FGF23^C-tail^ may be a potential therapeutic candidate for diabetic nephropathy.

## Additional file

Additional file 1: **Figure S1.** The effects of different doses of FGF23C-tail on the renal-functions in db/db mice. A: blood urea nitrogen (BUN); B: serum creatinine (CREA); C: microalbumin (mALB). (DOCX 144 kb)

## Abbreviations

BUN: Blood urea nitrogen
CTGF: Connective tissue growth factor
FGF23: Fibroblast growth factor 23
FGFR: Fibroblast growth factor receptor
GAPDH: Glyceraldehyde 3-phosphate dehydrogenase
HE: Hematoxylin and eosin
IF: Immunofluorescence
IHC: Immunohistochemistry
IL-6: Interleukin-6
IP10: Interferon-inducible protein-10
MCP-1: Monocyte chemotactic peptide-1
NF-kB: Nuclearfactor-kappaB
PAI-1: Plasminogen activator inhibitor-1
PAS: Periodic acid-Shiff
TEM: Transmission electron microscopy
TNFα: Tumor necrosis factor α
TNF-β1: Tumor necrosis factor-beta1
αSMA: A-smooth muscle actin
