# Supplementary material for: FGF23C-tail improves diabetic nephropathy by attenuating renal fibrosis and inflammation
Source: BMC Biotechnol. 2018 May 30;18:33. doi: 10.1186/s12896-018-0449-7 (PMC5975516; doi:10.1186/s12896-018-0449-7)
Supplement: Supplementary file 1 — Figure S1. The effects of different doses of FGF23C-tail on the renal-functions in db/db mice. A: blood urea nitrogen (BUN); B: serum creatinine (CREA); C: microalbumin (mALB). (DOCX 144 kb) [file 12896_2018_449_MOESM1_ESM.docx]

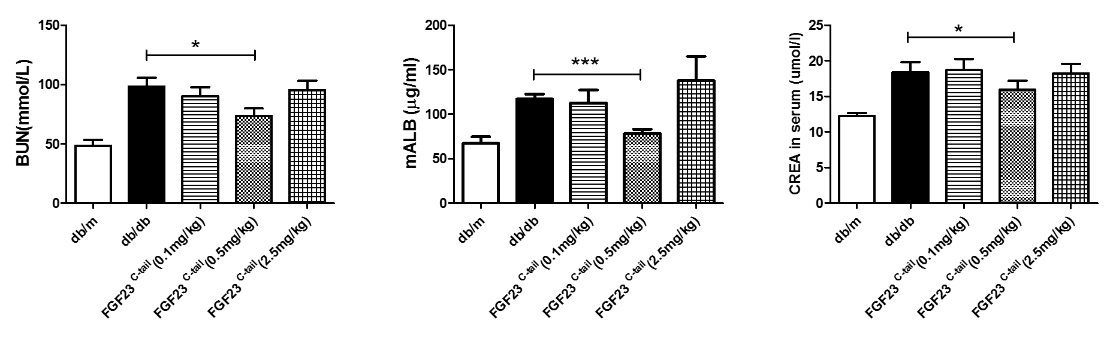


Figure S1 The effects of different doses of FGF23^C-tail^ on the renal-functions in db/db mice.
A: blood urea nitrogen(BUN); B: serum creatinine(CREA); C: microalbumin (mALB)
